# Practice-relevant model validation: distributional parameter risk analysis in financial model risk management

**DOI:** 10.1007/s10479-022-04574-x

**Published:** 2022-03-04

**Authors:** Mark Cummins, Fabian Gogolin, Fearghal Kearney, Greg Kiely, Bernard Murphy

**Affiliations:** 1 Irish Institute of Digital Business, Dublin City University, Dublin 9, Ireland; 2 Leeds University Business School, University of Leeds, Leeds, LS2 9JT UK; 3 Queen’s Management School, Queen’s University Belfast, Riddel Hall, Belfast BT9 5EE UK; 4 Gazprom Marketing and Trading Limited, 20 Triton St, London, NW1 3BF UK; 5 Kemmy Business, School, University of Limerick, Limerick, Ireland

**Keywords:** Risk management, Model validation, Parameter risk, Distributional analysis, Natural gas storage modelling

## Abstract

An objective of model validation within organisations is to provide guidance on model selection decisions that balance the operational effectiveness and structural complexity of competing models. We consider a practice-relevant model validation scenario where a financial quantitative analysis team seeks to decide between incumbent and alternative models on the basis of parameter risk. We devise a model risk management methodology that gives a meaningful distributional assessment of parameter risk in a setting where market calibration and historical estimation procedures must be jointly applied. Such a scenario is typically driven by data constraints that preclude market calibration only. We demonstrate our proposed methodology in a natural gas storage modelling context, where model usage is necessary to support profit and loss reporting, and to inform trading and hedging strategy. We leverage our distributional parameter risk approach to devise an accessible technique to support model selection decisions.

## Introduction

The topic of model risk, and model uncertainty more broadly, has commanded considerable attention across a diverse range of operations research and quantitative finance contexts. For example, healthcare network design (Denoyel et al. 2017); logistics and transportation (Koks et al. 2015); hazardous waste processors (Spear et al. 1994; Cooke 2009; Piegorsch 2014); environmental risk assessors, health and safety, and engineering (Alexander and Sarabia 2012); climate change modelling (Reis and Shortridge 2020); and financial services and insurance firms (Huang et al. 2010; Barrieu and Scandolo 2015; Coqueret and Tavin 2016; Alexander and Chen 2019). In the financial services industry, model use is widespread for trading, investment and hedging purposes (Aloui et al. 2014; Atil et al. 2014; Choukroun et al. 2015). Therefore, there is a need to understand, quantify and manage the associated model risks. Indeed, an external impetus for model risk management practice among financial institutions comes from regulatory and supervisory oversight. Regulatory authorities in the US and the EU have set out guidelines to create a common framework that outlines how financial institutions should manage model risk, with reference to organisational culture and governance, and best practice approaches to the activity of model validation. Recent operations research model risk studies with a similar finance application focus to ours include Huang et al. (2010), Barrieu and Scandolo (2015) and Coqueret and Tavin (2016). Firstly, Huang et al. (2010) demonstrate how their framework supports the selection of a robust portfolio of financial investments that addresses parameter estimation uncertainty by incorporating rarely observed worst-case scenarios. Secondly, Barrieu and Scandolo (2015) illustrate how applications of model risk can support compliance with regulatory requirements, including the Basel framework for assessing financial capital adequacy. Finally, Coqueret and Tavin (2016) consider the problem of model risk for variance swaps and forward-start options.

In this study, we consider a practice-relevant model validation scenario where an incumbent model exists, and is being appraised from a model risk perspective against one or more alternative, competing models.^1^ More specifically, we assume a model validation team working in an energy company that seeks to appraise a new model that is being proposed to replace an incumbent model. The model is assumed to be applied for deriving model-based valuation and mark-to-model measurement for a complex non-traded natural gas storage contract.The importance of modelling storage in energy markets is highlighted by Abid et al. (2019), Gaïgi et al. (2019), Weibelzahl and Märtz (2020) and Goutte et al. (2021), with Halvorsen-Weare and Fagerholt (2013) and Ameur et al. (2020) highlighting some of the considerations specific to natural gas storage.^2^ Storage has become a prominent feature of the natural gas markets. This is primarily due to large variation in winter and summer natural gas requirements driven by heating demand fluctuations. A gas storage contract allows a holder to buy gas to inject into storage during summer months when it is relatively cheap and to withdraw and sell the gas the following winter when prices are higher, with a key driver in determining the value of such natural gas storage contracts being the correlation matrix associated with the term structure of natural gas forward prices. Using these storage forward contracts allows market participants to smooth out seasonal demand fluctuations and to increase market liquidity (Le Fevre 2013), as well as providing a high degree of return certainty to an organisation considering investing in storage facilities.

Model risk considerations are important in the context of valuing storage contracts given the high materiality associated with such contracts. However, the current model risk literature offers very few studies that consider energy market applications. Bannor et al. (2013) were the first to focus on this issue where they investigate the parameter risk associated with a power plant valuation model. In the case of gas storage valuation, Hénaff et al. (2018) estimate the parameter risk associated with two proposed price models using only historical information for estimation. The spot gas processes of Hénaff et al. (2018) exhibit both mean reversion and price spikes, both stylised features of the energy markets. In accounting for price spikes within the models used by Hénaff et al. (2018), the processes could be used to calibrate the model to the options market. However, this was not an approach followed by the authors nor was there any discussion of option pricing under the proposed models. Our work can therefore be viewed as an extension of the work of Hénaff et al. (2018) by analysing storage model parameter risk with reference to a flexible multifactor Mean Reverting Variance Gamma (MRVG) model specification that is *both* forward curve consistent and calibrated to market traded options.

The choice of price models at our disposal is limited due to the fact the there is little in the literature that approaches the storage valuation problem with a view to maintaining consistency with the volatility smile displayed by vanilla natural gas futures-options. Therefore, although price models developed for storage valuation have the ability to capture the forward curve time-spread dynamics well, for example Parsons (2013) and Boogert and De Jong (2011), they are generally driven by diffusion processes and thus fail to replicate the volatility smile observable in gas option markets. Conversely, traditional price models that are capable of replicating the smile, such as the stochastic volatility model (Heston , 1993) or the SABR model (Hagan et al. , 2002), yield perfectly correlated forward curve returns and therefore capture little or no extrinsic value to the storage. Many of the extant gas storage models are inadequately specified to effectively capture the excess kurtosis evident in natural gas market returns, with model performance typically compromised in the pursuit of simpler solutions to the optimisation problem that underlies gas storage valuation. The multifactor MRVG model, in contrast, offers notable benefits in capturing excess kurtosis and time spread volatility, whilst maintaining consistency with the vanilla options market, and offering computationally efficient pricing solutions, such as Fourier transform based methods (Cummins et al. 2018). The question that arises is whether this improvement in model specification exacerbates parameter risk exposure.

Tunaru (2015) provides a comprehensive review of approaches to model risk analysis. Gupta and Reisinger (2011) provide a detailed introduction to the topic of calibration risk, including the key risk drivers and potential remedies. For example, the authors demonstrate how Bayesian averaging over potential parameter values can be used to both infer the level of calibration risk and also “smooth” the inverse problem associated with market calibration. Deryabin (2012) further extends this distributional view of calibration risk by introducing upper and lower bounds on the risk, which rely only upon the specification of the underlying model and its calibration to market data. This work was further extended by Bannör and Scherer (2013), who show how a meaningful distributional assessment of the parameter risk inherent in a given derivatives pricing model can be conducted using empirical densities, referred to as push forward densities, derived from the associated calibration errors and sample parameter variance. This approach marks a step-change in the quantification of calibration risk by providing a complete probabilistic framework for parameter risk assessment.

Our proposed method leverages Bannör and Scherer (2013) by imposing a distributional form on the feasible model parameter space and propagating this to the model derived valuations of the natural gas contract structure. We seek, however, a method that lends itself to joint calibration-estimation, which is a common scenario faced in practice where there is insufficient options market data to estimate all model parameters using a single calibration procedure. In our natural gas market setting, for instance, price models cannot be calibrated to options prices alone as there is insufficient liquidity in the calendar spread options market to capture implied forward price correlations, instead forcing the estimation of correlations directly from historical forward prices. Therefore, we extend the Bannör and Scherer (2013) methodology to a joint parameter calibration-estimation setting, where the combined use of underlying futures curve data (for the estimation procedure) and associated options market data (for the calibration procedure) is required.

We deliver some important messages for practitioners working in the model validation space within financial institutions. Firstly, we argue that the proposed distributional-based approach to model risk measurement provides a more complete probabilistic assessment of model risk exposure. The parsimonious distributional analysis of calibration-estimation parameter risk is more informative than previously proposed methods, such as, for instance, conventional sensitivity analysis (see, for example, Nalholm and Poulsen (2006)) or worst-case analysis (see, for example, Cont (2006) and Huang et al. (2010)). Our approach is also more appropriate than model averaging (see, for example, Bunnin et al. (2002)), given that the incumbent model has to be consistently used for a variety of purposes within the organisation. Furthermore, Cont (2006) observes that the purpose of risk management is not to predict prices but to quantify risk and so model averaging is less appropriate.

Secondly, we present guidance on how the distributional-based approach to model risk measurement allows a model validator to identify parameter combinations where extreme values are derived, particularly on the downside, and to quantify an associated likelihood of observing such parameter combinations. This supports model validators in setting model usage restrictions, identifying the market conditions where a model may need to be used more cautiously or suspended temporarily, or an alternative model specification may need to be deployed. As a form of sensitivity analysis, it allows model validators to identify and understand these parameter spaces (equivalently, market conditions) of interest from a model risk perspective. Residual benefits include assistance for traders in hedging more effectively against future market movements and insights for trade control functions to more effectively manage market risk exposure within the mark-to-model process.

Thirdly, we demonstrate how the distributional assessment approach to model risk measurement may be used by model validators to chose between alternative model specifications, reconciling the trade-off between model performance and model complexity. Specifically, we propose a simple model selection rule that works on a pairwise model basis. The approach we outline is informal in the sense that it focuses on the relative levels of parameter risk between competing models. It provides practitioners, however, with an accessible model selection technique that exploits the information provided by the distributional analysis of parameter risk.

The remainder of the paper is organised as follows. In Sect. 2, we introduce the parameter risk measurement methodology of Bannör and Scherer (2013) and derive our extension of this to a joint calibration-estimation setting. Section 3 presents our candidate models, providing the technical descriptions for the multifactor MRVG model in particular. In Sect. 4, we present the findings of our empirical storage model risk analysis, and we derive our model selection technique based on parameter risk. Section 5 concludes the paper.

## Model risk analysis

### Calibration risk measurement

In a calibration setting, Bannör and Scherer (2013) show how a meaningful distributional assessment of the parameter risk associated with a given derivatives pricing model can be determined when that derivatives pricing model is applied for the valuation of some exotic derivative contract. We consider a typical setting whereby before applying a parametric pricing model to value an exotic options contract, the model is first calibrated against a sample of liquid options contracts, returning point estimates for the model parameters. This ensures that the exotic options contract is valued in a way that is consistent with the observable options market. The calibration setting, however, presents a particular problem because the distribution of the parameter space is unavailable. Bannör and Scherer (2013) therefore devise a distribution construction that is consistent with the calibration procedure. The approach they advocate is to first calculate the calibration errors for a set of model parameter combinations $\theta \in \varTheta$, contained within some bounded parameter region $B_{\theta }$. The calibration errors might be defined, for example, as the root mean squared errors between the theoretical model derived option prices and the observed market option prices. The next step is to construct a probability distribution *R* of the model parameter space using a defined mapping of the calibration errors. The distribution on the model parameter space constructed in this way then allows for, what are termed, *push forward densities* to be constructed for the exotic option price. To this end, Bannör and Scherer (2013) define a transformation function *h*, which maps the calibration error, $\varepsilon \left( \theta \right)$, to the real line and possesses the following important properties: (i) *h* is decreasing, ensuring that parameter combinations which yield a higher total error are given less likelihood; and (ii)$\int h\left( \varepsilon \left( \theta \right) \right) d\theta =1$, ensuring the function meets the definition of a probability measure. One such transformation function put forward by Bannör and Scherer (2013) is the normal transformation function:

1 $$\begin{aligned} h\left( \varepsilon \left( \theta \right) \right) \equiv c\exp \left( -\left( \frac{\varepsilon \left( \theta \right) -\varepsilon ^{*}\left( \theta \right) }{\lambda }\right) ^{2}\right) \,\,\,\varepsilon \left( \theta \right) \ge \varepsilon ^{*}\left( \theta \right) \end{aligned}$$

with scaling parameters $\lambda >0$ and $c>0$ chosen so that the function returns a probability distribution centered on the minimum obtained calibration error $\varepsilon ^{*}\left( \theta \right)$, as achieved by the optimisation routine. This transformation then allows for easy construction of the push forward density of exotic option prices given that each parameter combination $\theta$ induces a mapping from a given calibration error, $\varepsilon \left( \theta \right)$, to an associated exotic option price, $V\left( X\left( \theta \right) \right)$.

Two practical advantages of the Bannör and Scherer (2013) approach are worth highlighting. Firstly, the approach provides a complete probabilistic framework to assess model risk exposure, whereby distributional characteristics can be readily evaluated. This provides greater insights for model validators into the trade-off between model performance and model risk. Secondly, the mapping of calibration errors to exotic option valuations through the push forward procedure means that a model validator can readily appraise those parameter combinations that result in extreme valuations, particularly on the downside. Indeed, the probabilistic framework allows a model validator to assign a likelihood to such extreme valuations and, hence, the likelihood of the associated parameter combinations. Relating such parameter combinations to meaningful economic market conditions means that a model validator can now set constraints and limitations on the use of a given model within the organisation.

### Joint calibration-estimation risk measurement

The model risk measurement approach of Bannör and Scherer (2013) is suitable in a pure calibration setting. However, such a setting is not always feasible. In this section we propose an extension of the Bannör and Scherer (2013) approach, which allows us to jointly assess combined market calibration and historical estimation parameter risk.^3^

We begin by denoting a general model of the forward curve that is dependent upon market calibrated parameters $\theta _{m}$ and historically estimated parameters $\theta _{h}$, such that $\theta =\left\{ \theta _{m},\theta _{h}\right\}$ defines the full set of model parameters. We generalise the transformation function, given in Eq. (1), such that it is capable of incorporating the additional historical information:

2 $$\begin{aligned} h\left( \varepsilon \left( \theta \right) \right) =h_{m}\left( \varepsilon \left( \theta \right) \,|\theta _{h}\right) p(\theta _{h}). \end{aligned}$$

The function $h\left( \varepsilon \left( \theta \right) \right)$ has the same properties as set out in Sect. 2.1 but is now decomposed into the product of $h_{m}\left( \varepsilon \left( \theta \right) \,|\theta _{h}\right)$, the *conditional market calibration error density* conditioned on the historically estimated parameters $\theta _{h}$, and the *historical sampling error density* $p(\theta _{h})$, associated with the historical estimation.

For the conditional market calibration error density, we utilise the normal transformation functional form, as described previously, i.e.

$$\begin{aligned} h_{m}\left( \varepsilon \left( \theta \right) \,|\theta _{h}\right) =c\exp \left( -\left( \frac{\varepsilon \left( \theta \right) -\varepsilon ^{*}\left( \theta \right) }{\lambda }\right) ^{2}\right) \,\,\,\varepsilon \left( \theta \right) \ge \varepsilon ^{*}\left( \theta \right) , \end{aligned}$$

centered on the minimum obtained calibration error $\varepsilon ^{*}\left( \theta \right)$, with scaling parameters $\lambda >0$ and $c>0$. The calculation of this conditional density proceeds by means of defining the calibration errors over a discretisation of the market calibrated parameters $\theta _{m}$, holding the historically estimated parameters $\theta _{h}$ fixed.

For the historical sampling error density $p(\theta _{h})$, we know from Bannör and Scherer (2013) that the maximum likelihood estimators of the parameters $\theta _{h}$ calculated from a sample of *n* observations are asymptotically normally distributed. This gives us a suitable functional form for $p(\theta _{h})$ - namely, the Gaussian density, which once scaled such that it integrates to unity can be used to give relative weight to the calibration induced probabilities.

To derive a functional form for $p(\theta _{h})$, we appeal to the, so called, ’delta method’ of Bannör and Scherer (2013). The key steps taken are as follows:

1. derive the sample covariance matrix, *C*, of the relative maturity forward curve returns;
2. using the sample Fischer Information matrix associated with *C*, derive the parameter covariance matrix, $\Sigma$;
3. derive the gradient of the model parameter estimation function, $\nabla g^{-1}$, where the estimation function takes the matrix *C* as input and returns a vector of model parameters;
4. derive the sample model parameter covariance matrix given by, $\left( \nabla g^{-1}\right) ^{'}\cdot \Sigma \cdot \nabla g^{-1}$; and
5. using the storage value parameter sensitivities $\nabla E_{\theta _{0}}$, derive the parameter risk variance.

If we take the payoff on a gas storage contract to be given by *X*, then collecting these steps gives

3 $$\begin{aligned} p(\theta _{h})\sim {\mathcal {N}}\left( 0,\left( \nabla E_{\theta _{0}}\left[ X\right] \right) ^{'}\cdot \left( \nabla g^{-1}\right) ^{'}\cdot \Sigma \cdot \nabla g^{-1}\cdot \nabla E_{\theta _{0}}\left[ X\right] \right) . \end{aligned}$$

This last result gives the storage value distribution induced by the uncertainty, represented by $\Sigma$, of the forward curve covariance matrix. The relationship between the two can be understood as firstly, weighting the matrix $\Sigma$ by the sensitivity of the model parameters to the forward curve covariance matrix, represented by $\nabla g^{-1}$, and then secondly, weighting the result by the sensitivity of the storage value to the model parameters, represented by $\nabla E_{\theta _{0}}$.

This approach retains the practical advantage for model validators as outlined in the previous section; namely, a complete probabilistic framework through which to assess model risk exposure, and the ability to identify parameter spaces where extreme downside valuations are likely, which assists the process of setting constraints and limitations on model usage.

## Gas storage valuation problem

From a commercial point of view, storage assets allow a trader to buy gas to inject into storage during summer months when it is relatively cheap and withdraw and sell the gas the following winter when prices are higher, thereby collecting the price difference as profit. Given a liquid forward market, traders have the ability to lock in a base value of the storage asset by locking in prices for future traded volumes. As such, organizations considering investment in a storage facility are aware, to a high degree of certainty, what return they will receive. This valuation and trading approach is referred to as *intrinsic valuation* and, as is the case for standard financial products, gives a lower bound on the storage value. Although such a method for valuing and operating the storage facility has the obvious benefit of eliminating market risk, it does so at a cost, that is, reducing completely the flexibility of the trader to adjust their planned injection/withdrawal schedule to respond to favourable market conditions. The prices at which storage capacity is offered in the market will typically exceed the intrinsic value and thus traders require a method to determine what extrinsic value can be extracted from the asset through dynamically trading in the underlying forward and options market. For this reason, it is much more common for practitioners to augment the intrinsic valuation approach with more advanced techniques.

We set up the gas storage valuation problem as per Cummins et al. (2018). As we are dealing with a physical storage infrastructure, we need to factor in standard physical constraints and operating characteristics. Let the current gas inventory level be given by $I$ $\in [I_{min},I_{max}]$. The amount of gas that can be injected or withdrawn from the storage asset in a given period is typically constrained and may be dependent upon both the time period and current inventory level. Let the injection and withdrawal rates be $i\left( t,I\right)$ and $w\left( t,I\right)$, respectively. Given a valuation period of length *T*,  we note the following constraints on the operation of the storage:

1. The allowed injection/withdrawal nomination times over the valuation period belong to a discrete set $\{t_{j}\}$.
2. For a given time step $t_{j}$ and inventory level *I*, the range of attainable storage levels is given as
  $$\begin{aligned} \left[ \right. \max \left( I-w\left( t_{j},I\right) ,I_{min}\right) ,\min \left( I+i\left( t_{j},I\right) ,I_{max}\right) \left. \right] \end{aligned}$$

We assume that when operating a storage asset the objective is to maximize the expected discounted cashflows arising from one’s injection/withdrawal policy. If we denote the cashflow derived from moving from storage level *I* to $I^{*}$, i.e. injecting or withdrawing, at a gas price $g_{t}$ as $a\left( I^{*};I,g_{t}\right)$, then, beginning at the end of the contract, the algorithm moves backwards in time to derive a solution for the initial storage value at time $t_{0}$ given by:

$$\begin{aligned} V\left( g_{t_{0}},I\right) =\sup _{I^{*}}a\left( I^{*};I,g_{t_{0}}\right) +E\left[ V\left( g_{t_{0}+\triangle t},I\right) |g_{t_{0}}\right] . \end{aligned}$$

The first term is the optimal cash flow at time $t_{0}$ from the injection/withdrawal decision and the second term is the expected value of the storage value over the time step $\Delta t$, given the prevailing gas price $g_{t_{0}}.$

We assume a basic “20in/20out” storage contract, whereby it takes 20 days to inject to full capacity from empty (*in*) and 20 days to empty once full (*out*). The deal commences immediately on the options quote date and lasts for a period of one year. We consider the UK’s NBP natural gas market, sourcing the benchmark options (in effect swaptions) for calibration from Bloomberg with a quote date of $19^{th}$ December 2012. We use the six-month (June 2013) and one-year (December 2013) maturity options at moneyness levels of $95\%,97.5\%,100\%,102.5\%$ and $105\%$. This sample is selected as it represents a data period that optimally showcases our approach given the lower levels of liquidity observed in gas swaption markets at this time.^4^ The data set used to estimate the historical covariance matrix is also sourced from Bloomberg and contains end of day prices for each business day in the period $18^{th}$ December 2009 to $19^{th}$ December 2012. The quoted prices used for estimation include day-ahead, month-ahead and quarterly contracts covering periods ending no later than one year from the observation date.

### Price model specifications

Before presenting the multifactor MRVG model specification, we first present two benchmark single factor models. The first of these models is the one-factor MRVG model (which we label MRVG-1F) as described by Cummins et al. (2017). The log-spot price model is specified by the following dynamics:

4 $$\begin{aligned} dx(t)&= \left( \frac{\partial f(0,t)}{\partial t}-\kappa _{j}\left( e^{-\alpha t}\right) +\alpha f(0,t)\right. \nonumber \\&\quad \left. -\alpha \int _{0}^{t}\kappa _{j}\left( e^{-\alpha (t-s)}\right) ds-\alpha x(t)\right) dt+dX\left( t\right) \end{aligned}$$

where *f*(0, *t*) is the initial log forward price at time *t*, $\alpha$ is the mean reversion rate, and *dX*(*t*) is a driftless Variance-Gamma process parameterised by $\sigma$, the process volatility, and $\nu$, the variance of the jump sizes. $\kappa _{j}$ is the cumulative of the Variance-Gamma process given by $\kappa _{j}\left( u\right) =-\frac{1}{v}\ln \left( 1-\frac{\sigma ^{2}v}{2}u^{2}\right)$. Eq. (4) implies a log-forward curve model of the form

$$\begin{aligned} df\left( t,T\right) =-\kappa _{j}\left( e^{-\alpha (T-t)}\right) dt+e^{-\alpha (T-t)}dX\left( t\right) . \end{aligned}$$

This model can be calibrated directly to market option prices and thus the parameter set distribution *R* can be obtained using the approach of Bannör and Scherer (2013), as set out in Sect. 2.1. The model is specified such that the instantaneous variance of different maturities along the forward curve shows exponential decay as time to maturity increases. This property, known commonly as the Samuelson Effect Serletis (1992), is a stylised feature of many commodity markets and particularly natural gas. For two log-forward prices, $f\left( t,T_{1}\right)$ and $f\left( t,T_{2}\right)$, the instantaneous covariance of returns is given by

$$\begin{aligned}&E\left[ \left( df\left( t,T_{1}\right) -E\left[ df\left( t,T_{1}\right) \right] \right) \left( df\left( t,T_{2}\right) -E\left[ df\left( t,T_{2}\right) \right] \right) \right] \\&\quad =\exp \left( -\alpha \left( T_{1}+T_{2}-2t\right) \right) \sigma ^{2}dt, \end{aligned}$$

which we can integrate to derive a terminal covariance over a given time horizon $\left( 0,t\right) ,$

$$\begin{aligned} cov(0,t)=\frac{\sigma ^{2}}{2\alpha }\left( \exp \left( -\alpha \left( T_{1}+T_{2}-2t\right) \right) -\exp \left( -\alpha \left( T_{1}+T_{2}\right) \right) \right) . \end{aligned}$$

Scaling by the square root of the returns variance for each maturity therefore yields the following measure of the terminal correlation:

$$\begin{aligned} \rho (0,t)=\frac{\left( \exp \left( -\alpha \left( T_{1}+T_{2}-2t\right) \right) -\exp \left( -\alpha \left( T_{1}+T_{2}\right) \right) \right) }{\sqrt{\left( \exp \left( -2\alpha \left( T_{1}-t\right) \right) -\exp \left( -2\alpha T_{1}\right) \right) \left( \exp \left( -2\alpha \left( T_{2}-t\right) \right) -\exp \left( -2\alpha T_{2}\right) \right) }}. \end{aligned}$$

Therefore, the mean reversion rate of the process, $\alpha$, which captures the exponential decay of forward price volatility with respect to maturity, fully controls the structure of the correlation among different points on the curve. This results in non-parallel shifts in the forward curve due to changes in the underlying stochastic driver. It is this dynamic forward curve movement that is crucial for storage valuation as it motivates the operator to exercise their optionality to switch planned injection and withdrawals thus creating extrinsic value. The variance of the jump magnitudes, $\nu$, controls the implied volatility smile attenuation and ensures that the model is consistent with the initial volatility surface.^5^

The second benchmark model considered is the one-factor Mean Reverting Jump Diffusion model (which we label MRJD) first specified by Deng (2000) and later used for swing contract valuation by Kjaer (2008):

5 $$\begin{aligned} dx(t)= & {} \left( \frac{\partial f\left( 0,t\right) }{\partial t}-\frac{1}{2}\sigma ^{2}+\frac{1}{4}\sigma ^{2}\left( 1-\exp \left( -2\alpha t\right) \right) -\kappa _{j}\left( \exp \left( -\alpha t\right) \right) \right. \nonumber \\&\quad \left. +\alpha f\left( 0,t\right) -\alpha \int _{0}^{t}\kappa _{j}\left( \exp \left( -\alpha \left( t-s\right) \right) \right) ds-\alpha x\left( t\right) \right) dt\nonumber \\&\qquad +\sigma dW\left( t\right) +dJ\left( t\right) . \end{aligned}$$

This model specification is similar to Eq. (4) with the only difference being the choice of stochastic drivers; $dW\left( t\right)$, a standard Brownian Motion, and *dJ*(*t*), a compound Poisson jump process driven by a symmetric double exponential distribution, that is, $J\left( t\right) =\sum _{i=1}^{N\left( t\right) }y_{i}$ where $N\left( t\right)$ is a Poisson process with arrival rate $\lambda$ and *y* is a random variable with the probability density function $f\left( y\right) =\frac{1}{2\mu }\left( \exp \left( -\frac{y}{\mu }\right) 1_{\left\{ y\ge 0\right\} }+\exp \left( \frac{y}{\mu }\right) 1_{\left\{ y<0\right\} }\right)$. Thus, as in the MRVG-1F model, the presence of the mean reversion parameter $\alpha$ is the primary driver of the storage value in controlling the forward curve covariance structure. The parameters relating to the jump-diffusion are $\sigma$, the diffusion volatility, $\lambda$, the jump arrival rate, and $\mu$ which controls the size of both positive and negative jumps. The jump diffusion specification is what allows us to replicate the volatility smile using this model, in a similar manner to the MRVG-1F model.^6^

This leads us to the specification of the flexible two-factor Mean Reverting Variance Gamma model (which we label MRVG-2F), as described by Cummins et al. (2018), which is a convenient extension of the MRVG-1F model. The inclusion of the second factor allows more flexibility in modelling the covariance structure of the forward curve and therefore should produce storage values more representative of the underlying dynamics. The dynamics of the log forward price under the MRVG-2F model are given by

$$\begin{aligned} df(t,T)=\frac{\partial f(0,T)}{\partial t}+dy^{(1)}(t,T)+dy^{(2)}(t,T), \end{aligned}$$

where

6 $$\begin{aligned} dy^{(1)}(t,T)= & {} \left( -\kappa _{j^{(1)}}be^{-\alpha \left( T-t\right) }\sigma \right) dt\nonumber \\&\quad +be^{-\alpha \left( T-t\right) }\sigma dX(t),\nonumber \\ dy^{(2)}(t,T)= & {} -\frac{1}{2}\left( e^{-\epsilon \left( T-t\right) }c\sigma \right) ^{2}dt\nonumber \\&\quad +e^{-\epsilon \left( T-t\right) }c\sigma dW(t), \end{aligned}$$

and *dW*(*t*) is a standard Brownian Motion and *dX*(*t*) is a Variance-Gamma process with unit volatility and jump size variance $\nu$. The first factor, which accounts for the majority of the forward curve variability, is a Mean Reverting Variance Gamma process. As with the MRVG-1F model, the main parameters for this factor can be calibrated to the options market. The parameters *b* and *c* represent the proportion of total variance attributed to the first and second factors respectively and would need to be estimated from the historical data. Letting $t\rightarrow T$ in Eq. (6), we can see that the volatility of the log spot gas price is in fact given by $\sigma \sqrt{b^{2}+c^{2}}$. The second factor is specified such that it approximates the typical shape of the sensitivity, which we refer to as the *volatility function*, of the forward curve to the second principal component of the forward curve returns covariance matrix. The parameters relating to the second factor can be estimated directly from the eigenvector values. The parameter $\epsilon$ controls the decay of the volatility function as maturity increases. The slope of the volatility function will have a direct impact on the covariance of different maturities along the forward curve. A sharply decaying curve will decrease the covariance between prompt forward prices and the back of the curve, which will lead to greater time-spread variance and thus higher storage value. As with the MRVG-1F model we can derive the instantaneous returns covariance for two log forward prices $f\left( t,T_{1}\right)$ and $f\left( t,T_{2}\right)$,

$$\begin{aligned}&E\left[ \left( df\left( t,T_{1}\right) -E\left[ df\left( t,T_{1}\right) \right] \right) \left( df\left( t,T_{2}\right) -E\left[ df\left( t,T_{2}\right) \right] \right) \right] \\&\quad =\exp \left( -\alpha \left( T_{1}+T_{2}-2t\right) \right) b^{2}\sigma ^{2}dt++\exp \left( -\epsilon \left( T_{1}+T_{2}-2t\right) \right) c^{2}\sigma ^{2}dt, \end{aligned}$$

with terminal covariance given by

$$\begin{aligned} cov(0,t)= & {} \frac{\left( \sigma b\right) ^{2}}{2\alpha }\left( \exp \left( -\alpha \left( T_{1}+T_{2}-2t\right) \right) -\exp \left( -\alpha \left( T_{1}+T_{2}\right) \right) \right) ,\\&+\frac{\left( \sigma c\right) ^{2}}{2\epsilon }\left( \exp \left( -\epsilon \left( T_{1}+T_{2}-2t\right) \right) -\exp \left( -\epsilon \left( T_{1}+T_{2}\right) \right) \right) . \end{aligned}$$

With $\epsilon \gg \alpha$, the MRVG-2F model, as it encompasses the MRVG-1F model, will attribute more value to a storage asset through this additional decorrelation of the forward curve returns.

In the above we assume independence between the sources of randomness. This restriction to independent processes does not prohibit us from capturing the inter-maturity pairwise dependency of forward curve returns to a high level of accuracy. Further, it allows us to specify the effect of each state variable on the forward curve dynamics independently, which is in agreement with traditional principal component based analysis of forward curve movements. Utilizing PCA in multifactor model specifications is common in energy markets; for example, see Clewlow and Strickland (1999).

The crucial model risk question we address with our joint calibration-estimation methodology is: to what extent should this additional value be discounted on the basis of the historical estimation risk inherent in the model estimation?

### Calibration and estimation procedures

The calibration procedure for estimating the parameters of both the MRVG-1F and MRJD models involves minimising the usual root mean squared error (RMSE) between model derived and market observed prices, formally,

$$\begin{aligned} \min _{\theta _{m}}\sqrt{\frac{1}{n}\sum _{i=1}^{n}\left( C_{i}^{model}\left( \theta _{m}\right) -C_{i}^{market}\right) ^{2},} \end{aligned}$$

where for the MRVG-1F model $\theta \equiv \theta _{m}=\left\{ \alpha ,\sigma ,\nu \right\}$, and for the MRJD model $\theta \equiv \theta _{m}=\left\{ \alpha ,\sigma ,\lambda ,\mu \right\}$, and *n* is the sample size. In order to calculate the model option prices, $C_{i}^{model}\left( \theta _{m}\right)$, we use the Fourier transform based swaption pricing algorithm developed by Cummins et al. (2018).

For the MRVG-2F model, we take a different approach due to the requirement to jointly calibrate and estimate the model parameters using market and historical data. From the full parameter set $\theta \equiv \left\{ \alpha ,\sigma ,\nu ,b,c,\epsilon \right\}$, we define the market calibrated parameter set as $\theta _{m}\equiv \left\{ \alpha ,\sigma ,\nu \right\}$, determining the shape of the volatility term structure of the first factor of the model, and we define the historically estimated parameters set as $\theta _{h}\equiv \left\{ b,c,\epsilon \right\}$, determining the shape of the volatility term structure of the second factor of the model. We utilise the market implied moment matching technique of Cummins et al. (2018), which involves firstly, inferring the moments of the underlying forward contracts from the prices of quoted options and secondly, calibrating the known model forward price moments to these values. In our exercise here, we focus on matching the first four moments of the forward prices underlying our option quotes. The historically estimated parameters of the MRVG-2F model are calculated by matching the entries of the eigenvectors given by spectral decomposition of the forward curve returns covariance matrix.

## Gas storage model risk analysis

The procedure for estimating calibration risk outlined by Bannör and Scherer (2013) begins by discretising the parameter space and then evaluating the pricing error to benchmark instruments for each parameter combination. Thus they reduce the multidimensional parameter space to a vector of error terms. Given that a large majority of these errors would fall outside of what would be deemed a reasonable calibration, the authors propose discarding parameter combinations that return an error greater than some cut-off point. In their numerical examples, Bannör and Scherer (2013) use a cut-off for the error term, given by the RMSE, of $2\%$ above the minimum calibration error achieved. Obviously the choice of cut-off will depend upon the average bid-offer percentage spread observed in the options market. For our analysis, we have chosen a cut-off of $3\%$ in order to reflect the relatively lower liquidity of natural gas options.

One could in theory, search over a dense and wide discretisation of the parameter space in order to identify multiple regions of local minima. However, there is a computational demand when proceeding in this manner, which necessitates the need for a coarser and narrower discretisation. This is particularly true when calibrating to non-standard option contracts, as is the case here for the NBP swaptions contracts. We therefore suggest, first searching for local minima by selecting the initial parameter values in ones search function from a wide and relatively sparse discretisation of the parameter space. Once each of these local minima have been identified one can construct a region encompassing each in order to identify local calibration risk. For each of the models specified above, the calibration to our options data, using the simplex method of Lagarias et al. (1998), did produce multiple local minima dependent upon the choice of initial parameters. However, in each case the local minimum either yielded an RMSE far in excess of our acceptable cut-off or returned parameter values similar to our optimal values. Therefore we will focus only on a reduced parameter space encompassing our initial parameter estimates.

We focus first on the benchmark MRVG-1F and MRJD models, which require estimation from market data only. Following the example of Bannör and Scherer (2013), we choose $h\left( \cdot \right)$ to be the normal transformation given by Eq. (1). For the scaling parameter, $\lambda$, we use the sample variance of the calibrated errors centered on the minimum value and chose *c* such that the density values sum to unity. For both the MRVG-1F and MRJD models, the mean reversion rate, $\alpha ,$ and the instantaneous volatility, $\sigma$, determine the term structure of volatility at different maturities along the forward curve. As outlined in Sect. 3, this exponentially decreasing term structure acts to decorrelate the forward curve returns at different maturities and is the primary driver of the extrinsic storage value. The variance of the jump sizes in the forward curve returns are controlled by the parameter $\nu$ in the MRVG-1F model and by $\lambda$ and $\mu$ in the MRJD model. The main purpose of these parameters is to aid in matching the implied volatility smile present in the natural gas options market. The calibrated parameters are given in Table 1. The associated RMSE is $1.07\%$ for the MRVG-1F model, while the RMSE is slightly higher for the MRJD model at $1.10\%$.

**Table 1:** MRVG-1F/MRJD models: calibrated model parameters

MRVG-1F	$\alpha$	$\sigma$	$\nu$		RMSE
	*0.2162*	*0.201*	*0.2560*		$1.07\%$
MRJD	$\alpha$	$\sigma$	$\lambda$	$\mu$	RMSE
	*0.2099*	*0.0334*	8.7966	0.047	$1.10\%$

MRVG-1F denotes the one-factor Mean Reverting Variance Gamma (MRVG-1F) model of Cummins et al. (2017), as described in Eq. (4). The parameters are as follows: $\alpha$ is the mean reversion rate; $\sigma$ is the volatility of the Variance Gamma process; and $\nu$ is the variance of the jump sizes of the Variance Gamma process. MRJD denotes the Mean Reverting Jump Diffusion (MRJD) model of Deng (2000), as described in Eq. (5). The parameters are as follows: $\alpha$ is the mean reversion rate; $\sigma$ is the volatility of the diffusion process; $\lambda$ is the arrival rate of the jump process; and $\mu$ controls the size of both positive and negative jumps under the jump process.

**Fig. 1:**
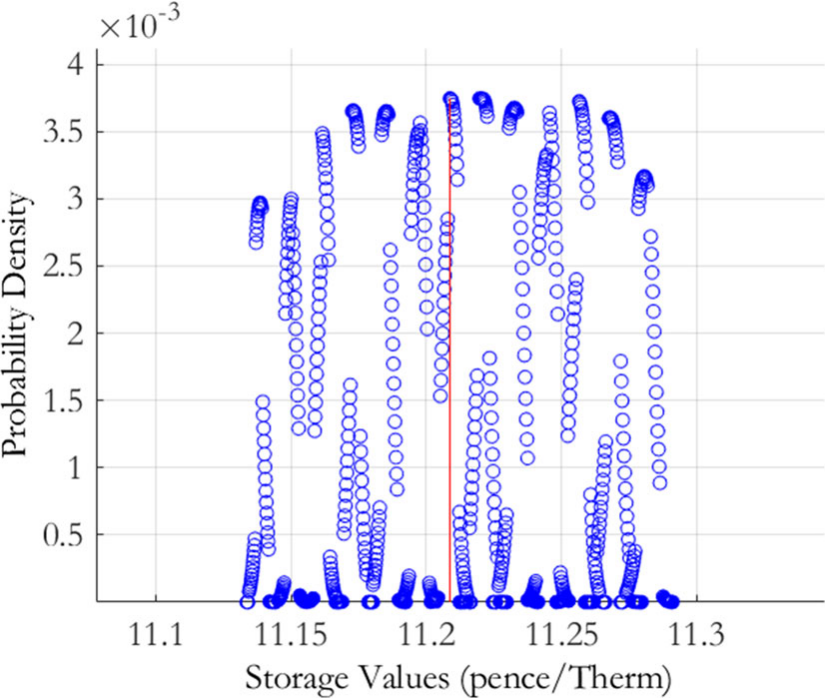
MRVG-1F storage value push forward density

To investigate the local risk of our initial calibration of the MRVG-1F model, we select mean reversion $\alpha$ values ranging from $70\%$ to $130\%$, in steps of $5\%$, of the calibrated estimate. For the volatility $\sigma$ and the variance of jump magnitudes $\nu$, we use a tighter range of values from $90\%$ to $110\%$, in steps of $1.5\%$, of the calibrated estimates, which reflects the greater sensitivity of the RMSE to changes in these two parameters. In total, this yielded 2, 548 distinct parameter combinations. We next iterate over this parameter space and discard any parameter combinations that produce RMSE measures greater than our $3\%$ limit. The resulting parameter space reduced to 807 parameter combinations. Using the error terms associated with these parameters, we derive an error density as per Eq.(1) and then evaluate the storage value at each point in this reduced parameter space. The resulting calibration risk induced storage value push forward density is displayed graphically in Fig. 1 and the distributional characteristics are given in Table 2. As is evident from the graph, the density appears almost uniform over the acceptable calibration range. The apparent presence of columns of points in Fig. 1 correspond to storage values at each $\alpha$ point in the parameter space. This behaviour can be rationalised by observing that changes in $\alpha$ have little effect on the calibration error relative to changes in $\sigma$ and $\nu$ due to the sensitivity of the implied volatility smile to changes in model volatility and kurtosis. This means that a much wider range of $\alpha$ values fall within the acceptable parameter space. Further, due to the sensitivity of storage values to the model covariance structure, the choice of $\alpha$ will impact the value much more than both $\sigma$ and $\nu$.

**Fig. 2:**
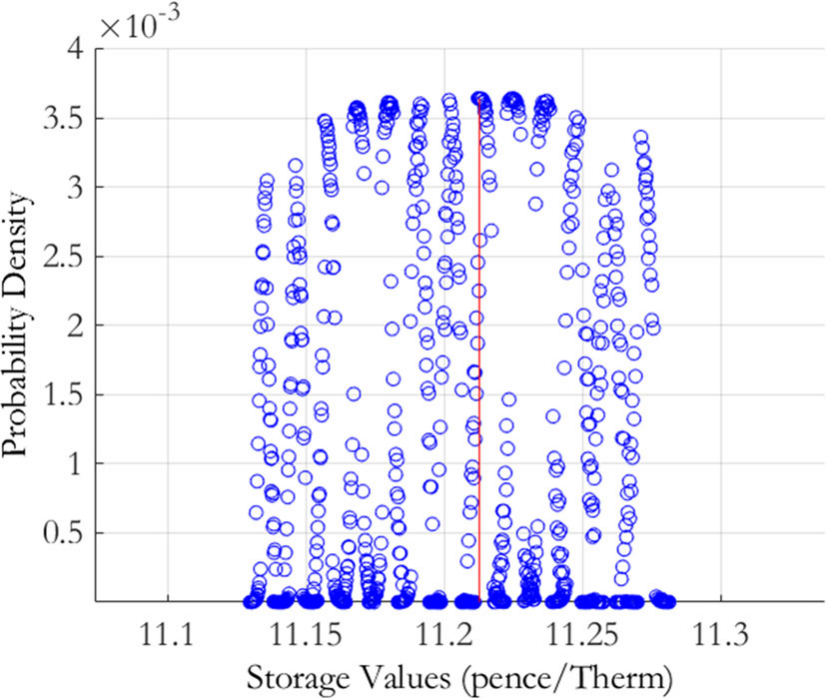
MRJD storage value push forward density

For the MRJD model, we again select the mean reversion $\alpha$ range to be $70\%$ to $130\%$, in steps of $5\%$, of the calibrated estimate. The values for the volatility $\sigma$, jump arrival rate $\lambda$ and jump size $\mu$ were selected to range from $90\%$ to $110\%$, in steps of $4\%$, of the initial calibration estimates. This tighter range is reflective of the greater sensitivity of the RMSE to changes in these parameters. In total this gave 2, 808 distinct parameter combinations, of which 795 were within the cut-off threshold of 3. The resulting push forward storage density is displayed in Fig. 2 and the distributional characteristics are again given in Table 2. As we can observe visually, and also from the summary statistics, there is not much difference between the push forward densities from both models. The MRVG model returns a slightly higher expected value than the MRJD model but also has a slightly higher level of variability. It would appear on the basis of this analysis that both models carry almost equivalent levels of calibration risk. A difference lies however in the skewness of the storage value push forward densities determined by the MRVG-1F and MRJD models, the former indicating a negatively skewed distribution and the latter a positively skewed distribution.

The plot presents the storage value push forward density for the MRVG-1F model of Eq. (4), derived using the calibration risk measurement procedure of Bannör and Scherer (2013) as set out in Sect. 2.1. The procedure applies the following normal transformation function to the calibration errors $\varepsilon \left( \theta \right) ,$ defined under the discretisation of the model parameter space $\theta \equiv \theta _{m}=\left\{ \alpha ,\sigma ,\nu \right\}$:

$$\begin{aligned} h\left( \varepsilon \right) :=h_{\lambda }^{{\mathcal {N}}}\left( \varepsilon \right) =c\exp \left( -\left( \frac{\varepsilon -\varepsilon ^{*}}{\lambda }\right) ^{2}\right) \,\,\,\varepsilon \ge \varepsilon ^{*}, \end{aligned}$$

with scaling parameters $\lambda >0$ and $c>0$ chosen so that the function returns a probability distribution centered on the minimum obtained calibration error $\varepsilon ^{*}$, as achieved by the optimisation routine employed. For the implementation, we chose $\lambda =7.9358e-05$ and $c=266.92$.

For the discretisation of the model parameter space, we chose values for the mean reversion rate $\alpha$ ranging from $70\%$ to $130\%$, in steps of $5\%$, of the calibrated estimate, and values for the volatility $\sigma$ and variance of jump sizes $\nu$ ranging from $90\%$ to $110\%$, in steps of $1.5\%$, of the respective calibrated estimates.

**Table 2:** MRVG-1F/MRJD storage value distribution characteristics

	MRVG-1F	MRJD
Modus	11.2087	11.2127
Expected value	11.2116	11.2039
Coefficient of variation	0.38%	0.36%
Skewness	-0.034	0.021

MRVG-1F denotes the one-factor Mean Reverting Variance Gamma (MRVG-1F) model of Cummins et al. (2017), as described in Eq. (4). MRJD denotes the Mean Reverting Jump Diffusion (MRJD) model of Deng (2000), as described in Eq. (5)

The plot presents the storage value push forward density for the MRJD model of Eq. (5), derived using the calibration risk measurement procedure of Bannör and Scherer (2013) as set out in Sect. 2.1. The procedure applies the following normal transformation function to the calibration errors $\varepsilon \left( \theta \right) ,$ defined under the discretisation of the model parameter space $\theta \equiv \theta _{m}=\left\{ \alpha ,\sigma ,\lambda ,\mu \right\}$:

$$\begin{aligned} h\left( \varepsilon \right) :=h_{\lambda }^{{\mathcal {N}}}\left( \varepsilon \right) =c\exp \left( -\left( \frac{\varepsilon -\varepsilon ^{*}}{\lambda }\right) ^{2}\right) \,\,\,\varepsilon \ge \varepsilon ^{*}, \end{aligned}$$

with scaling parameters $\lambda >0$ and $c>0$ chosen so that the function returns a probability distribution centered on the minimum obtained calibration error $\varepsilon ^{*}$, as achieved by the optimisation routine employed. For the implementation we chose $\lambda =8.2115e-05$ and $c=274.70$.

For the discretisation of the model parameter space, we chose values for the mean reversion rate $\alpha$ ranging from $70\%$ to $130\%$, in steps of $5\%$, of the calibrated estimate, and values for the volatility $\sigma$, the jump arrival rate $\lambda$, and the jump size $\mu$, ranging from $90\%$ to $110\%$, in steps of $4\%$, of the respective calibrated estimates.

### Joint calibration-estimation risk

The MRVG-2F model, as set out in Eq. (6), requires calibration to options market data and also parameter estimation from historical futures market data. The initial market calibrated and historically estimated model parameters are given in Table 3. As in the MRVG-1F case, the market calibrated parameters contain the mean reversion rate $\alpha$ and volatility $\sigma$ which determine the term structure of volatility of the first factor, along with the jump size variance $\nu$, which will determine the attenuation of the model implied volatility smile. The historically estimated parameters *b* and *c* act to weight each factor by their contribution to overall forward curve variation, thus the variance of the log spot gas price will be simply $\sigma ^{2}\left( b^{2}+c^{2}\right)$. The mean reversion rate $\epsilon$ of the second factor determines the slope of that factor’s term structure of volatility. This has the effect of introducing returns of a much higher absolute magnitude for short term maturities and therefore acts to decorrelate the overall forward curve returns.

**Table 3:** Calibrated-estimated model parameters: MRVG-2F model

$\theta _{m}$			$\theta _{h}$			
$\alpha$	$\sigma$	$\nu$	*b*	*c*	$\epsilon$	RMSE
0.1148	0.2518	0.1675	0.7511	0.6254	12.734	$1.86\%$

MRVG-2F denotes the two-factor Mean Reverting Variance Gamma model of Cummins et al. (2018), as described in Eq. (6). The first factor is specified by a Mean Reverting Variance Gamma process, whereby $\alpha$ is the mean reversion rate, $\sigma$ is the volatility and$\nu$ is the jump size variance of the process. $\theta _{m}\equiv \left\{ \alpha ,\sigma ,\nu \right\}$ is the market calibrated parameter set, determining the shape of the volatility term structure of the first factor. The parameters *b* and *c* represent the proportion of total variance attributed to the first and second factors respectively. The second factor is specified such that it approximates the typical shape of the sensitivity, i.e. the *volatility function*, of the forward curve to the second principal component of the forward curve returns covariance matrix. The parameter $\epsilon$ controls the decay of the volatility function as maturity increases. $\theta _{h}\equiv \left\{ b,c,\epsilon \right\}$ is the historically estimated parameters set, determining the shape of the volatility term structure of the second factor

**Table 4:** MRVG-2F storage value distribution characteristics

	$h_{m}\left( \varepsilon \left( \theta \right) \,|\theta _{h}\right)$	$h_{m}\left( \varepsilon \left( \theta \right) \right) p(\theta _{h})$
Modus	15.4117	15.4117
Expected value	15.2558	15.2528
Coefficient of variation	0.69%	2.17%
Skewness	0.0033	0.0001

Following the joint calibration-estimation risk measurement procedure set out in Sect. 2.2, $h_{m}\left( \varepsilon \left( \theta \right) \,|\theta _{h}\right)$ is the conditional market calibration error density and $h_{m}\left( \varepsilon \left( \theta \right) \right) p(\theta _{h})$ is the specific form of the joint calibration-estimation risk density $h_{m}\left( \varepsilon \left( \theta \right) \,|\theta _{h}\right) p(\theta _{h})$, where independence is assumed between the calibration error and the values of the parameters estimated from historical data

**Fig. 3:**
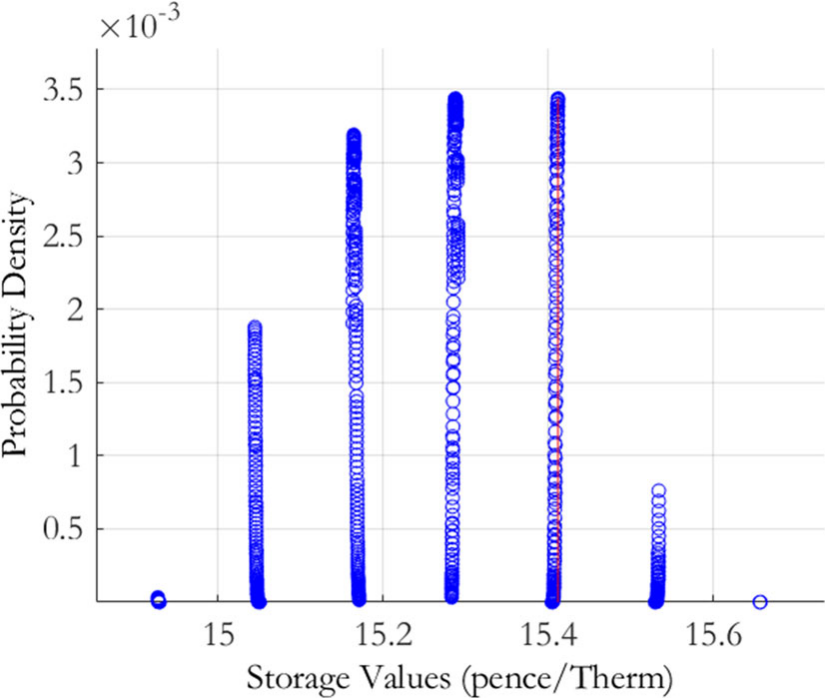
MRVG-2F conditional market calibration error density

Recall from Eq. (2) that the parameter risk induced storage value density, $h\left( \varepsilon \left( \theta \right) \right) ,$ for the MRVG-2F model is decomposed into the conditional market calibration error density $h_{m}\left( \varepsilon \left( \theta \right) \,|\theta _{h}\right)$ and the historical sampling error density $p(\theta _{h})$. We shall proceed by first determining the former density $h_{m}\left( \varepsilon \left( \theta \right) \,|\theta _{h}\right)$. We have chosen the same parameter space discretisation for $\theta _{m}$ as in the MRVG-1F case, that is, $\alpha$ values ranging from $70\%$ to $130\%$, in steps of $5\%$, of the calibrated estimate, and $\sigma$ and $\nu$ values ranging from $90\%$ to $110\%$ of their initial calibrated values, in steps of $1.5\%$. This returned 2, 548 parameter combinations, of which 838 were within the threshold of $3\%$ of the minimum calibration error. The conditional market calibration error density for storage value, $h_{m}\left( \varepsilon \left( \theta \right) \,|\theta _{h}\right)$, is displayed graphically in Fig. 3, with the distributional characteristics given in Table 4.

The plot presents the conditional market calibration error density $h_{m}\left( \varepsilon \left( \theta \right) \,|\theta _{h}\right)$ for the MRVG-2F model of Eq. (6), derived using the joint calibration-estimation risk measurement procedure set out in Sect. 2.2. The procedure applies the following generalised normal transformation function to the calibration errors $\varepsilon \left( \theta \right)$, defined under the discretisation of the model parameter space $\theta \equiv \left\{ \theta _{m},\theta _{h}\right\}$:

$$\begin{aligned} h\left( \varepsilon \left( \theta \right) \right) =h_{m}\left( \varepsilon \left( \theta \right) \,|\theta _{h}\right) p(\theta _{h}). \end{aligned}$$

The conditional market calibration error density is defined as follows: $h_{m}\left( \varepsilon \left( \theta \right) \,|\theta _{h}\right) =c\exp \left( -\left( \frac{\varepsilon -\varepsilon ^{*}}{\lambda }\right) ^{2}\right) \,\,\,\varepsilon \ge \varepsilon ^{*},$ centered on the minimum obtained calibration error $\varepsilon ^{*}$. For the implementation we chose $\lambda =7.7304e-05$ and $c=290.70$.

For the discretisation of the market parameter space, we chose values for the mean reversion rate $\alpha$ ranging from $70\%$ to $130\%$, in steps of $5\%$, of the calibrated estimate, and values for the volatility $\sigma$ and variance of jump sizes $\nu$ ranging from $90\%$ to $110\%$, in steps of $1.5\%$, of the respective calibrated estimates.

Comparing the conditional market calibration error density to the MRVG-1F and MRJD cases, we see that the value is much higher but also the coefficient of variation is almost double that of the single factor models and thus the confidence one would have in the calibrated value is lower. From the plot of the push forward density we observe a clustering of storage values at distinct levels. These points relate to different $\sigma$ values in the discretised parameter space. Recall that in the MRVG-1F and MRJD models, there was an apparent clustering at distinct $\alpha$ values due to the sensitivity of the storage value to changes in this parameter. For the MRVG-2F model however, the value will be primarily driven by the relatively high estimated $\epsilon$ value, which will act to decorrelate the near-dated and far-dated maturities on the forward curve due to the steep decline in the volatility term structure of the second factor. From the model specification given in Eq. (6), we can see that the level of this volatility term structure will be proportional to $\sigma$, which thus gives a rationale for the relatively large value sensitivity to this parameter and the storage value density behaviour observable in Fig. 3.

**Fig. 4:**
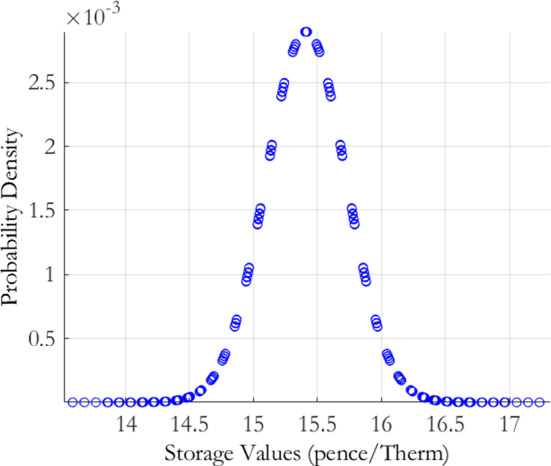
MRVG-2F historical sampling error density

We now proceed to derive the historical sample error density for the storage value, associated with the historically estimated parameters $\theta _{h}$. In order to derive the parameter estimation risk induced storage value density, we must first estimate the Jacobian of our storage value with respect to our model parameters $\theta _{h}$. We do so by first difference based numerical differentiation. Specifically, for each parameter $\vartheta _{h}\in \theta _{h}$, we calculate $\frac{\partial V}{\partial \vartheta _{h}}\approx \frac{V\left( \vartheta _{h}+\frac{1}{2}\eta \right) -V\left( \theta -\frac{1}{2}\eta \right) }{\eta }$, where *V* represents the initial storage value and $\eta$ is a small induced parameter perturbation. In our numerical implementation we choose $\eta =0.0001.$ The results of this are displayed in Table 5. The *c* parameter, which controls the percentage of spot variance accounted for by the second factor, has the greatest impact on the storage value, followed by the $\epsilon$ parameter, which is ultimately responsible for the extrinsic value attributable to the second factor. Intuitively, this can be understood as the $\epsilon$ parameter reducing the autocorrelation of the second factor and *c* being responsible for passing this decorrelation through into the forward curve returns. The *b* parameter has little impact on the storage value which is due to the low levels of extrinsic value attributable to the first factor. The historical sample error density for the storage value is shown in Fig. 4.

**Table 5:** MRVG-2F model: historical parameter deltas

Parameter	Delta
*b*	0.0039
*c*	8.8059
$\epsilon$	0.1402

Parameter sensitivity calculated using finite differences. Formally, for each parameter $\vartheta _{h}\in \theta _{h}$, we calculate $\frac{\partial V}{\partial \vartheta _{h}}\approx \frac{V\left( \vartheta _{h}+\frac{1}{2}\eta \right) -V\left( \theta -\frac{1}{2}\eta \right) }{\eta }$, where *V* represents the initial storage value and $\eta$ is a small induced parameter perturbation. In our numerical implementation we choose $\eta =0.0001$

The plot presents the historical sampling error density $p(\theta _{h})$ for the MRVG-2F model of Eq. (6), derived using the joint calibration-estimation risk measurement procedure set out in Sect. 2.2. The estimation risk induced storage value distribution, obtained by applying the delta method of Bannör and Scherer (2013), set out in Sect. 2.1, is described as follows:

$$\begin{aligned} \left( E_{\theta _{h}}\left[ X\right] -E_{\theta _{h,0}}\left[ X\right] \right) \sim {\mathcal {N}}\left( 0,\left( \nabla E_{\theta _{0}}\left[ X\right] \right) ^{'}\cdot \left( \nabla g^{-1}\right) ^{'}\cdot \Sigma \cdot \nabla g^{-1}\cdot \nabla E_{\theta _{0}}\left[ X\right] \right) , \end{aligned}$$

where $\Sigma$ represents the uncertainty of the forward curve covariance matrix, $\nabla g^{-1}$ represents the sensitivity of the model parameters to the forward curve covariance matrix, and $\nabla E_{\theta _{0}}$ represents the sensitivity of the storage value to the model parameters.

For the construction of the combined calibration and estimation risk induced storage value density, we make an assumption of independence between the calibration error and the values of the parameters estimated from historical data. This is primarily due to the computational effort involved in constructing the conditional calibration risk induced storage value density, which would grow exponentially if the density was a function of a second equally dense parameter space. This assumption reduces Eq. (2) to

$$\begin{aligned} h\left( \varepsilon \left( \theta \right) \right) =h_{m}\left( \varepsilon \left( \theta \right) \right) p(\theta _{h}). \end{aligned}$$

The distribution characteristics of this combined density are given in Table 4. While the greater flexibility of the MRVG-2F model captures more extrinsic value of the storage contract, relative to the MRVG-1F and MRJD models, this is at the cost of greater uncertainty as to its true value, with the variability of the storage value increasing dramatically due to exposure to historical estimation risk. Hence, there is higher risk of suffering P&L variations attributable to the choice of model parameters.

### Model comparison and selection

Now that we have evaluated the storage value push forward densities associated with the candidate models, we are positioned to discuss the actions that can be carried out using this information. We begin with the choice of appropriate risk-captured bid-offer prices to compensate for parameter risk exposure. The main motivation for this is to allow traders and trade control groups to reserve capital upfront to cover a potential loss due to the calibration and estimation of pricing models. To inform this decision, we suggest first constructing the cumulative distribution function (CDF) from the push forward density and then choosing percentiles appropriate to one’s level of risk tolerance as bid-offer prices.^7^

**Fig. 5:**
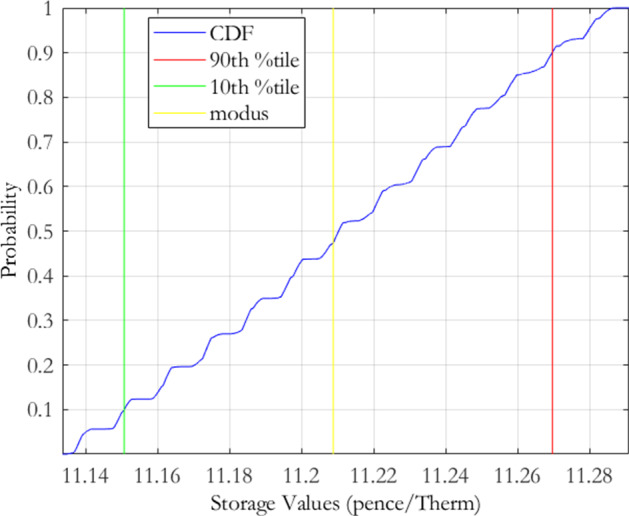
MRVG-1F storage value cumulative distribution function

**Fig. 6:**
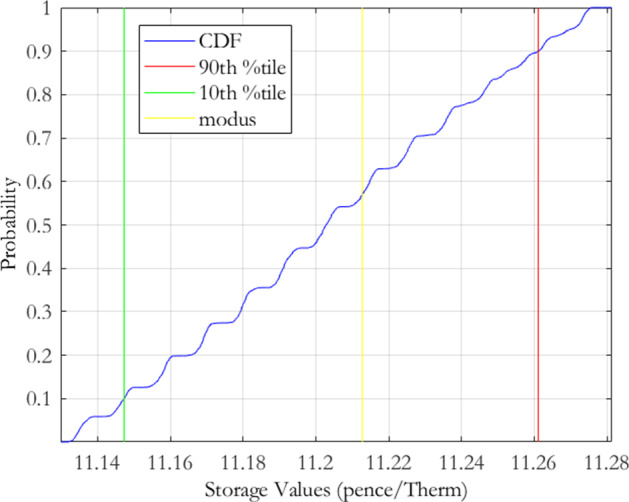
MRJD storage value cumulative distribution function

**Fig. 7:**
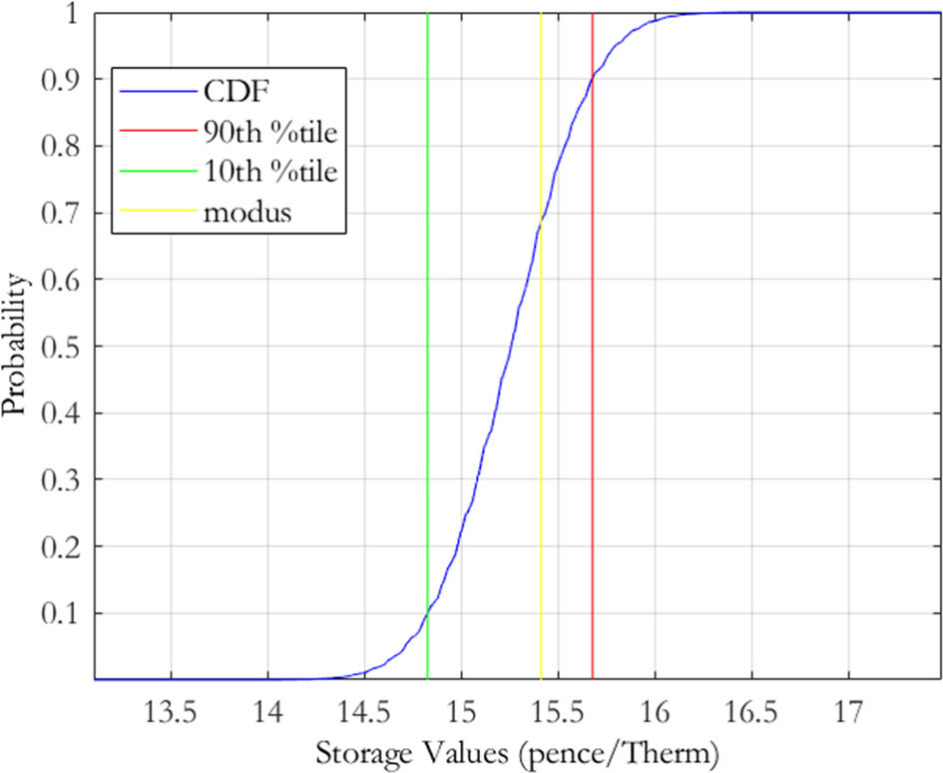
MRVG-2F storage value cumulative distribution function

Figures 5, 6 and 7 display the CDFs associated with the MRVG-1F, MRJD and MRVG-2F models respectively. In the case of the MRVG-1F model, taking the $10^{th}$ and $90^{th}$ percentiles as bid and offer levels gives us prices of 11.1507 and 11.2697 respectively, which equates to a spread of $1.06\%$ of the mean value. The MRJD CDF gives similar bid-offer prices of 11.1473-11.2611, which is a spread of $1.02\%$ of the mean value. The MRVG-1F bid-offer spread is marginally wider than that of the MRJD model, which is consistent with the previously reported results. In contrast, for the MRVG-2F model, the $10^{th}$ and $90^{th}$ percentiles of the CDF give bid-offer prices of 14.8266-15.6772, representing a much wider spread of $5.58\%$ of the mean value, over 5 times that of the MRVG-1F and MRJD models. Coinciding with our previous evidence, this of course reflects the much greater uncertainty around the true value of the storage contact under the MRVG-2F model, notwithstanding its ability to capture greater extrinsic value. A key question that arises from this is of course: is it possible to bear this greater parameter risk for the flexibility that the MRVG-2F model offers? This question provides our motivation to next develop a practical model selection technique.

Authorising the use of one model over another would typically fall on the model validation team within the risk control function of an organisation. The approach we outline here is informal in the sense that it focuses only on the relative levels of parameter risk and should be viewed as a complement to a wider model specification analysis. To frame the example, let us assume that the MRVG-1F model is the incumbent and the trading desk has requested the use of the MRVG-2F model for valuation and P&L reporting. Once a rigorous theoretical examination of the assumptions and inputs underpinning the model has been carried out, as per best practice model validation, there is then an obvious need for a quantitative methodology capable of appropriately ranking the models with respect to parameter risk. Comparing the valuations returned by both models in the context of the model risk they carry and for the products most relevant to the company is the main goal of such an analysis. Here, we utilise the Average-Value-at-Risk (AVaR), or equivalently, expected shortfall, based risk functional given by Bannör and Scherer (2013). AVaR is a coherent risk measure, defined for a given percentile $\alpha$ as

$$\begin{aligned} AVaR_{\alpha }\left( X\right)\equiv & {} \frac{1}{\alpha }\int _{0}^{\alpha }VaR_{b}\left( X\right) db, \end{aligned}$$

where $VaR_{b}\left( X\right)$ is the $b\%$ Value-at-Risk for a position with payoff *X*. The AVaR at $\alpha =100\%$ therefore corresponds to the expected value of a position under a given probability measure.^8^ Bannör and Scherer (2013) utilise AVaR as the underlying coherent risk measure in the risk functional, $R*AVaR_{\alpha }\left( X\right)$, defined as

$$\begin{aligned} R*AVaR_{\alpha }\left( X\right)\equiv & {} AVaR_{\alpha }\left( Q\rightarrow E_{Q}[X]\right) , \end{aligned}$$

where *R* is the distribution of the family of measures ${\mathcal {Q}}$. $R*AVaR_{\alpha }\left( X\right)$ captures the model risk quantified by the distribution *R*, which is isolated from the model-intrinsic risk within a specific model $Q\in {\mathcal {Q}}.$

**Fig. 8:**
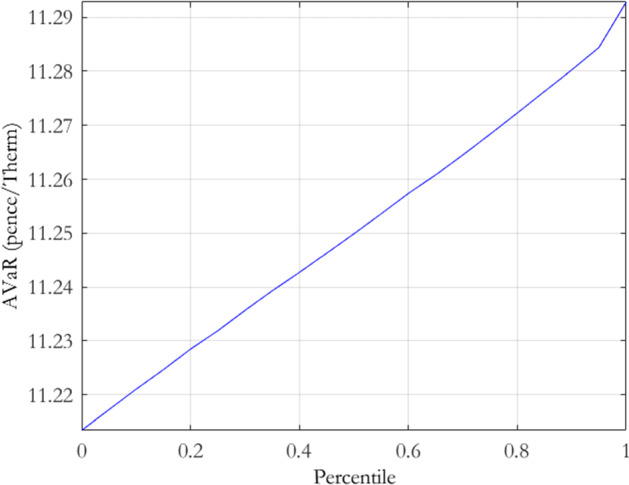
MRVG-1F storage risk-captured offer prices

**Fig. 9:**
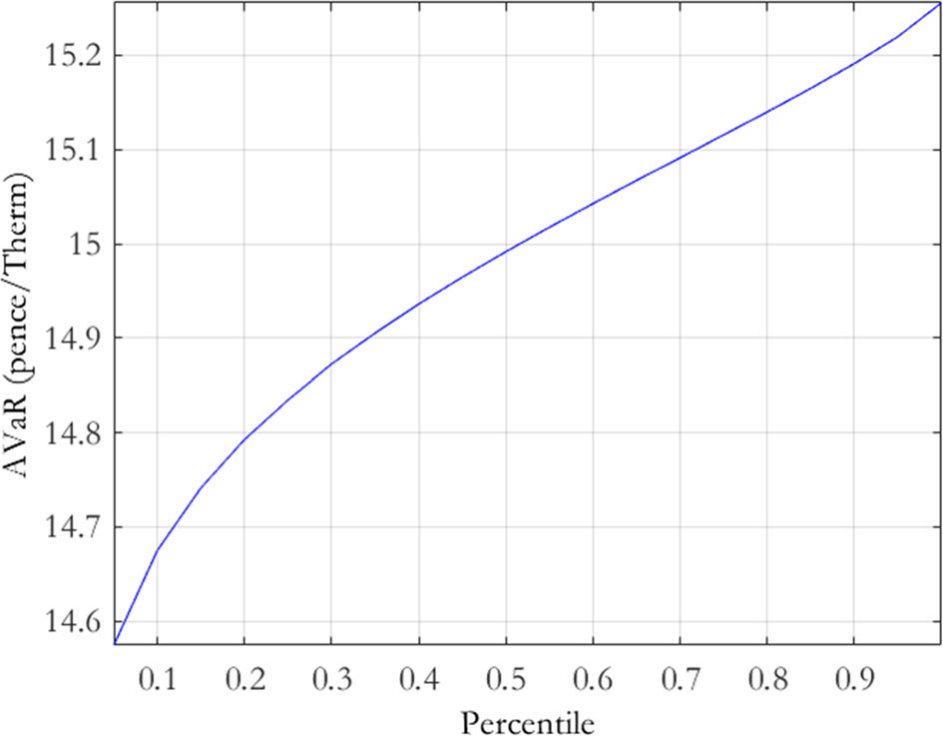
MRVG-2F storage risk-captured bid prices

In the case where one candidate model is being proposed as a potential replacement for an incumbent model and they rank equally in all other potential model comparison metrics, a naive approach would be to chose the model which returns the highest expected value on a given asset, ignoring the model risk implicit in the valuations. A conservative parameter risk based rule for switching would be to require the new candidate model to yield a bid price,$-R*AVaR_{\alpha }^{\text{ candidate }}\left( -X\right)$, at a chosen percentile to be greater than the offer price, $R*AVaR_{\alpha }^{\text{ incumbent }}\left( X\right)$, of the incumbent model. Not only does this guarantee that the model risk adjusted price levels in the new model exceed those of the incumbent, it also returns a minimum cash value associated with switching, i.e. the bid minus the offer. Figure 8 displays the risk-captured offer prices for the MRVG-1F model, while Fig. 9 displays the risk-captured bid prices for the MRVG-2F model, at varying percentile levels in the range $\left( 0,1\right]$. As the MRVG-2F risk-captured bid prices dominate the MRVG-1F offer prices at all percentiles, the resulting decision would be that the MRVG-2F model is an acceptable replacement for the MRVG-1F, as the increased parameter risk is sufficiently compensated.

This decision outcome is important. It was argued earlier that the MRVG-2F model allows more flexibility in modelling the covariance structure of the forward curve and therefore should produce storage values more representative of the underlying dynamics. The MRVG-2F model, however, increases the complexity of the model specification over the MRVG-1F model as it (i) moves from a one-dimensional state space to a two-dimensional state space and (ii) doubles the number of parameters to be estimated from three to six. However, we have just shown that despite the increased exposure to parameter risk that this brings, the ability to capture greater extrinsic value compensates sufficiently this model risk. Our study is therefore an important complement to the work of Cummins et al. (2018). While the latter study argues the merits of the MRVG-2F model in better modelling gas storage value, the authors ignore the issue of model risk exposure. We address this practitioner relevant problem directly in our study, providing a methodological innovation coupled with practical guidance for the model validation function within organisations.

## Conclusion

We consider a practice-relevant model validation scenario where a model validation team is seeking to decide between an incumbent and an alternative, competing model, on the basis of parameter risk. We contribute through an extension of the calibration risk measurement methodology of Bannör and Scherer (2013) where we incorporate both market calibration and historical estimation within a meaningful distributional assessment of parameter risk. This is a problem that is typical in practice, where due to data constraints, calibration to options contracts only is not feasible. We demonstrate our proposed methodology with an application to assess parameter risk in a natural gas storage modelling exercise, where complex price model dynamics are required to optimally capture the extrinsic value inherent in such contracts. We further contribute through leveraging our advocated distributional parameter risk analysis to devise an accessible model selection technique.

While we provide an important methodological contribution to deal with scenarios where joint calibration-estimation is required, we simultaneously deliver some important messages for model validators working in practice. Firstly, the distribution-based approach to model risk measurement that we advocate provides a sound probabilistic framework upon which to assess model risk exposure. Secondly, model validators can follow the model risk approach we take to better understand the sensitivity of complex derivative valuations to particular parameter combinations, aiding them to better set constraints and limitations to model usage within their organisations. Thirdly, the distributional assessment approach to model risk measurement may be readily leveraged by model validators as a decision support tool for model choice recommendations.

A useful direction for future research would be to leverage the model risk management analysis of this paper to produce a comprehensive study of the alternative storage valuation methods proposed in the literature. A significant challenge is that there is no consensus with regards to the approach used for gas storage modelling and valuation, instead we see a range of approaches advocated - spanning spot-based, forward-based and practitioner heuristic models. Careful consideration would additionally need to be given to the price model dimension and the optimization dimension of the gas storage valuation problem (see, for example, Bjerksund et al. (2011)). We see this as an important next step in the development of this topic.

## Footnotes

1 While Cont (2006) argues that the distinction between model risk and parameter risk is somewhat irrelevant, we adopt both terms in our discussion to aid clarity.

2 Trading in natural gas has increased significantly due to the development of liquid centralized trading hubs. The hubs act as single trading points for the transfer of gas entering and exiting their respective markets. We focus on the UK’s National Balancing Point hub, which was the first and most liquid of these centralized trading hubs. A derivatives market for forwards and futures contracts developed on the back of the centralized trading hubs, with the Intercontinental Exchange’s NBP futures contracts accounting for one third of total NBP trading volume (Heather 2012).

3 While we make a distinction between “estimation” and “calibration” here, it is important to note that liquid derivatives market contract prices, namely, futures contracts and plain vanilla options contracts, are used for the respective parameter search procedures. Both sources of data therefore inform us about the risk-neutral measure, *Q*. Our estimation context is therefore different to the situation where a parametric pricing model is fitted to underlying spot price data defined under the physical measure, *P*.

4 More recently, Aslam et al. (2020), Aloui et al. (2020), Goodell and Goutte (2020) and Mensi et al. (2020) highlight the impact of COVID-19 on crude oil and natural gas markets. This pandemic again highlights the importance of constructing model risk techniques that can cope with periods of reduced energy swaption liquidity.

5 Cummins et al. (2017) provide a technical discussion of the MRVG-1F model, including the derivation of the associated characteristic function and the application of Fourier transform methods for the pricing of gas storage capacity.

6 See Cummins et al. (2017) for more technical details of the MRJD model.

7 This approach can be seen as an informal, but consistent, alternative to the risk functional approach adopted by Bannör and Scherer (2013).

8 See Föllmer and Knispel (2011) for a comprehensive overview of the AVaR risk measure.
